# Distribution and diel vertical movements of mesopelagic scattering layers in the Red Sea

**DOI:** 10.1007/s00227-012-1973-y

**Published:** 2012-06-13

**Authors:** Thor A. Klevjer, Daniel J. Torres, Stein Kaartvedt

**Affiliations:** 1 King Abdullah University of Science and Technology, Thuwal, 23955-6900 Saudi Arabia; 2 Woods Hole Oceanographic Institution, MS #30, Woods Hole, MA 02543 USA

## Abstract

The mesopelagic zone of the Red Sea represents an extreme environment due to low food concentrations, high temperatures and low oxygen waters. Nevertheless, a 38 kHz echosounder identified at least four distinct scattering layers during the daytime, of which the 2 deepest layers resided entirely within the mesopelagic zone. Two of the acoustic layers were found above a mesopelagic oxygen minimum zone (OMZ), one layer overlapped with the OMZ, and one layer was found below the OMZ. Almost all organisms in the deep layers migrated to the near-surface waters during the night. Backscatter from a 300 kHz lowered Acoustic Doppler Current Profiler indicated a layer of zooplankton within the OMZ. They carried out DVM, yet a portion remained at mesopelagic depths during the night. Our acoustic measurements showed that the bulk of the acoustic backscatter was restricted to waters shallower than 800 m, suggesting that most of the biomass in the Red Sea resides above this depth.

## Introduction

Mesopelagic is a label usually attached to the so-called twilight zone of the water masses (Ramirez-Llodra et al. 2010), where surface light is still detectable during the daytime, but at very low levels compared with the epipelagic zone. This zone is usually considered to extend from about 200 to 1,000 m depth (Gjøsaeter and Kawaguchi 1980; Sutton et al. 2008). During the infancy of acoustic studies in the 1950s and 1960s, one of the major findings was how ubiquitous the mesopelagic deep scattering layers were (Moore 1950; Hersey et al. 1962). Many of the inhabitants of this zone carry out diel vertical migrations (DVM; Gjøsaeter and Kawaguchi 1980), residing at depth during the day and swimming towards the surface to feed at shallow depths only under the cover of darkness. DVM is today recognised as the biggest movement of biomass on earth (Hays 2003), with major consequences for ecology (Ramirez-Llodra et al. 2010) and biogeochemical cycling (Robinson et al. 2010).

Mesopelagic migrators are believed to be an important component of the biological pump, since they feed near the surface during the night and defecate at depth during day (Robinson et al. 2010). However, understanding of the absolute or even relative amount of carbon exported from this assemblage is unclear, as the biomass, behaviour and vertical distribution of mesopelagic animals remain poorly described. In terms of biomass, the most important mesopelagic migrators are probably mesopelagic fish, which typically are small and presently have limited commercial value (Gjøsaeter and Kawaguchi 1980). Mesopelagic fish in the Red Sea have seldom been studied [with the exception of (Dalpadado and Gjøsaeter 1987)], while the neighbouring Arabian Sea is home to the world’s largest stock of mesopelagic fish, which have been the subject of several studies (Gjøsaeter 1984; Venema 1984; Ashjian et al. 2002). The Arabian Sea is among the most productive marine areas in the world, which may account for the larger number of studies, whereas the Red Sea has traditionally been considered an oligotrophic area (Weikert 1982; Halim 1984) whose pelagic ecosystems have not attracted much research attention. In addition to being oligotrophic, the Red Sea is extremely warm, with temperatures remaining above 21.7 °C down to the bottom (Halim 1984). The combination of high temperature and oligotrophic conditions undoubtedly has a strong influence on the energy budgets of the mesopelagic animals.

In addition, a mid-water oxygen minimum zone is present in the Red Sea. The presence of subsurface layers of reduced oxygen content in the oceans has long been recognised (Longhurst 1967 and references therein) and extends over large areas of the open ocean (Childress and Thuesen 1995). The range of these oxygen minimum zones is predicted to expand with global warming (Stramma et al. 2010), which will likely have ecological consequences (Wishner et al. 2008).

In this paper, we evaluate the presence, distribution and diel vertical movement of the mesopelagic scattering layers in the extreme environment of the Red Sea. Low oxygen conditions may constrain distributions of both plankton and fish (Childress and Seibel 1998), but may also provide a refuge from predators (Prince and Goodyear 2006). While some mesopelagic fish appear to be tolerant of low oxygen waters (Childress and Seibel 1998), there are no studies at such high temperatures, and interactions between low oxygen concentrations and temperature have been found to affect the distribution and behaviour of other pelagic organisms (Tremblay et al. 2010). Significant daytime feeding is unlikely in the very low plankton concentrations at mesopelagic depths of the Red Sea (Wishner 1980; Weikert 1982), and high temperature suggests fast digestion after nocturnal feeding. How these factors affect the mesopelagic organisms in the Red Sea is unknown. Furthermore, because increasing temperature and decreasing oxygen levels are predicted for the oceans (Ramirez-Llodra et al. 2010; Stramma et al. 2010), the Red Sea mesopelagic ecosystem may be viewed as a potential model for the future of mesopelagic ecosystems.

## Materials and methods

### Cruise description

**Fig. 1:**
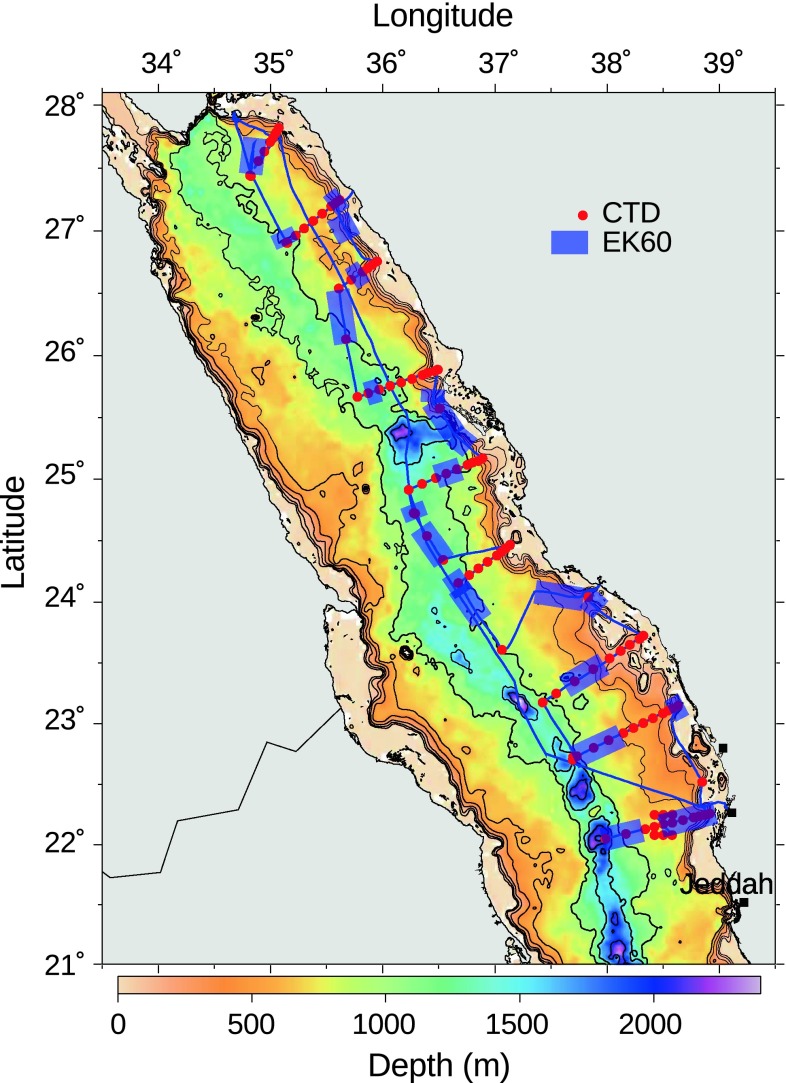
Map showing cruise track (*blue line*) and positions of the CTD casts (*red points*) in the Red Sea. The *blue transparent rectangles* indicate sections of the cruise track included in the EK60 data

Data were collected from the R/V “Aegaeo” in the northern Red Sea during cruises from March to May, 2010. Acoustic data were collected during a leg of the cruise stretching from the mouth of the Gulf of Aqaba to approximately Jeddah during the last half of March. This leg was conducted primarily to collect hydrographic data and consisted of a series of transects stretching out from the coast towards the deep centre of the Red Sea, interspersed by periods of steaming along the axis of the Red Sea (Fig. 1). Conductivity, temperature, and depth (CTD) casts were made at regular intervals with a SeaBird 9/11 CTD, which was also equipped with a SeaBird oxygen sensor and a WETLabs integrated chlorophyll-a/turbidity sensor. The oxygen profiles revealed zones with oxygen minima at mesopelagic depths. In this paper, our definition of the mesopelagic oxygen minimum zone (OMZ) corresponds coarsely to hypoxic water (oxygen values of <1.4 mL L^−1^, Ekau et al. 2010).

**Fig. 2:**
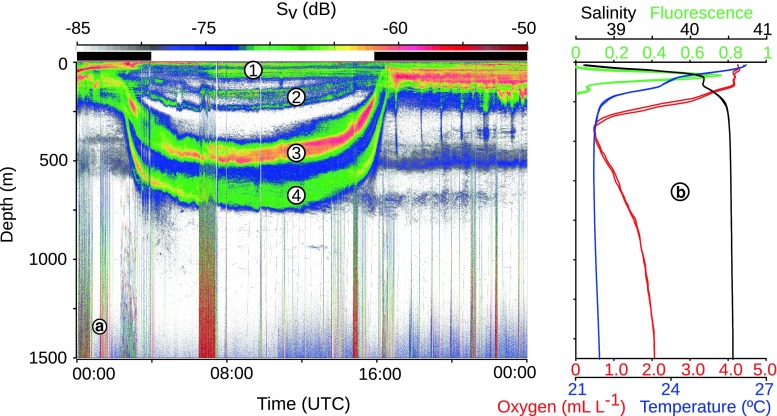
Echograms from April 27.** a** An entire 24 h period, with the different scattering layers indicated by *numbers*. Day and night periods are indicated with *white* and *black bars* above echogram.** b** The vertical profiles of salinity (*black line*), temperature (*blue line*), fluorescence (*green line*), and oxygen (*red line*) closest in time and space to the echogram in** a**

Additional acoustic data were collected in a later leg of the cruise that was conducted to study the deep brine pools (Antunes et al. 2011) of the area. These data were used in the echogram in Fig. 2, and also for the estimation of migration speeds of layers, but were otherwise not analysed.

Data for tentative identification of the acoustically observed organisms were obtained from daytime video recordings collected with a Remotely Operated Vehicle (ROV) (for description see Batang et al. 2012), as well as from observations during a dive with the manned submersible “Thetis.” These observations were used to assess the presence of different phylogenetic groups at different depths. These observations were only possible using artificial light, so both avoidance and attraction effects may have influenced the results.

### Echosounder

Continuous acoustic measurements were made with a Simrad EK60 echosounder, operating at a frequency of 38 kHz. We were unable to calibrate the hull-mounted EK60 system prior to the cruise, but the EK60 data were corrected prior to postprocessing based on a calibration performed in 2011. The echosounder data were episodically affected by noise from different sources, so prior to import into the LSSS software (Korneliussen et al. 2009) used for postprocessing, the data were subjected to a series of filters for removing bad data. These filters worked by comparing the integrated backscatter over a depth range with the background backscatter over the same depth range. Background intensities were detected using a median filter 400 pings wide, updated every 100 pings; pings affected by attenuation were defined as pings with backscatter more than 6 dB below the median in either of the depth ranges 50–600 m or 600–1,000 m. Periods where backscatter was more than 4 dB above background levels in the depth range 800–1,000 m were also marked. Pings tagged by these filters were excluded in further analyses. Lastly a simple 9-point running median (horizontal) removed shorter irregular spikes. This filter will introduce a bias in the data by removing the highest intensity data. The backscatter estimates are therefore conservative.

After manual scrutiny of the remaining data, residual background noise was removed by standard techniques (Korneliussen 2000), before the data were integrated in 2 min by 2 m bins at a threshold of −82 dB. For the analysis of daytime distribution, these bins were chosen from periods when the sound scattering layers had their deepest distribution. For the analysis of night-time distributions, bins were chosen from periods when the scattering layers did not display active migration behaviour. These acoustic data are presented along with oxygen and fluorescence data obtained from the CTD casts. The data that were closest in time to the acoustic data are overlaid on the profiles. The fluorescence and oxygen contours in the different plots are therefore similar, but not identical.

We used unfiltered/unprocessed echograms as a basis to describe vertical distribution of the scatterers. Vertical migration speeds were also computed from measurements of these echograms. Layer migration speeds were computed by recording the time it took the upper boundary of a layer to move 100 m vertically (respectively from 100 to 200 m depth and from 400 to 500 m depth).

### The Lowered Acoustic Doppler Current Profiler (LADCP)

One of the goals of the physical oceanographic survey was to determine the velocity structure along the transects. A dual 300 kHz Workhorse LADCP system mounted on the CTD was deployed in vertical casts to measure currents. Stations were closely spaced to capture narrow along-isobath currents and spanned the Saudi Arabian waters from the offshore reefs to the deep central part of the basin. For most stations, the LADCP was configured to measure twenty 8-meter bins at approximately 1 s per sample interval. This setup was designed to obtain the best possible velocity data. Generally speaking, insonifying a larger volume of water with larger bin sizes results in a lower standard deviation of velocity measurements at the expense of vertical resolution. The optimal LADCP setup for backscatter analysis would therefore be to use smaller bin sizes. However, valuable backscatter information can be derived from the described setup. The echo intensity data for each beam was saved as part of the regular recorded output of the ADCPs.

Hull-mounted ADCPs have been shown to be effective for observing zooplankton migration (Flagg and Smith 1989), but calibration of the echo intensity of an ADCP is difficult. Our use of the LADCP eliminates the need for range correction of the acoustic signal since the instrument is lowered through the water column. By using the same bin at the same distance from the ADCP (in this case from 14.2 to 22.2 m), we eliminated the need to correct for signal loss due to absorption and attenuation in our LADCP backscatter measurements.

## Results

**Fig. 3:**
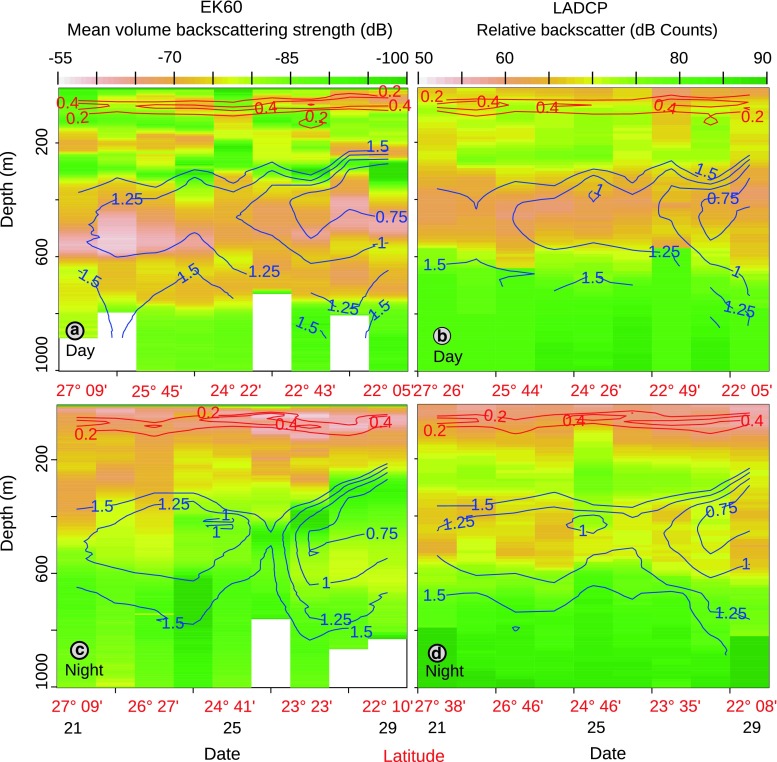
Level plots of acoustic biomass profiles (10–1,000 m). Days are plotted along the *x*-*axis*, with one observation per day (the latitudes of the CTD stations are given for every second date in *red*). The depth is plotted along the *y*-*axis*, and the *colours* correspond to amount of acoustic biomass. Absence of data is shown as *white* fields. Fluorescence (*red*) and oxygen (*blue*) concentrations are overlaid. *Upper panels* daytime data; *lower panels* night-time data*. Left column* EK60 data. *Right column* LADCP (300 kHz) data. In the EK60 plots, each *horizontal pixel*-*column* represents a daily profile. In the LADCP plots, each pixel *horizontally* represents a single CTD cast. For the EK60, the overlay values are from the CTD cast closest in time to the centre of the profile; in the LADCP plots, the overlay values are concurrent. *Blue lines* oxygen concentrations in mL L^−1^. The *contour lines* are 0.25 mL L^−1^ apart. *Red lines*: contours of fluorescence

Fluorescence values generally peaked between depths of 50 and 100 m (Figs. 2, 3). Some profiles also suggested a second peak deeper in the water column, at about 130 m. A weak trend of increasing fluorescence with decreasing latitude was suggested in the data, but the fluorescence generally showed similar variations within the same area as between north and south.

The oxygen profiles revealed a distinct oxygen minimum zone (OMZ) (Fig. 2). The depth of minimum oxygen decreased from ~450 m in the north to a depth of ~350 m further south. Minimum values of oxygen ranged from well above 1 mL L^−1^ in the northern part of the Red Sea to approximately 0.5 mL L^−1^ near the southern end of the cruise.

### Vertical distribution of volume backscatter (biomass)

At least four scattering layers were discernible in the 38 kHz acoustic data during the daytime, that is, on echograms and on vertical profiles of acoustic scattering (Figs. 2, 3). These scattering layers were detected throughout the north/south transect (bottom depth permitting) (Fig. 3).

Layer 1 was identified by increased backscatter in the upper 100 m. Layer 2, found in the depth interval of 150–300 m, had a tendency of separating into narrower sublayers. The sublayering occurred in relatively well-oxygenated waters and below the peak in fluorescence. Layer 3 was found in the depth interval 400–600 m. This layer occupied depths corresponding to the OMZ, although the peak backscatter from this layer occurred deeper than the minimum oxygen concentrations. We measured the migration velocity of this layer between 100 and 200 m, tracing the shallowest part of the layer. The average descent speed was 5.1 m min^−1^ (sd = 1, *n* = 20), and the average ascent speed was 2.9 m min^−1^ (sd = 0.6, *n* = 21). At 600–800 m deep, a weaker scattering layer (layer 4) was usually visible. We also measured the migration velocity of this layer between 400 and 500 m, tracing the shallowest part of the layer. The average descent speed was 4.3 m min^−1^ (sd = 0.7, *n* = 22), and the average ascent speed was 2.6 m min^−1^ (sd = 0.4, *n* = 16).

No defined layers of scatterers were evident in the 38 kHz data beneath these depths, suggesting that the density of larger animals below ~1,000 m was low (Fig. 2). However, the acoustic records revealed traces of single individual scatterers beneath these depths. Animals were therefore present, but at a much reduced density, with some individuals appearing to migrate vertically.

**Fig. 4:**
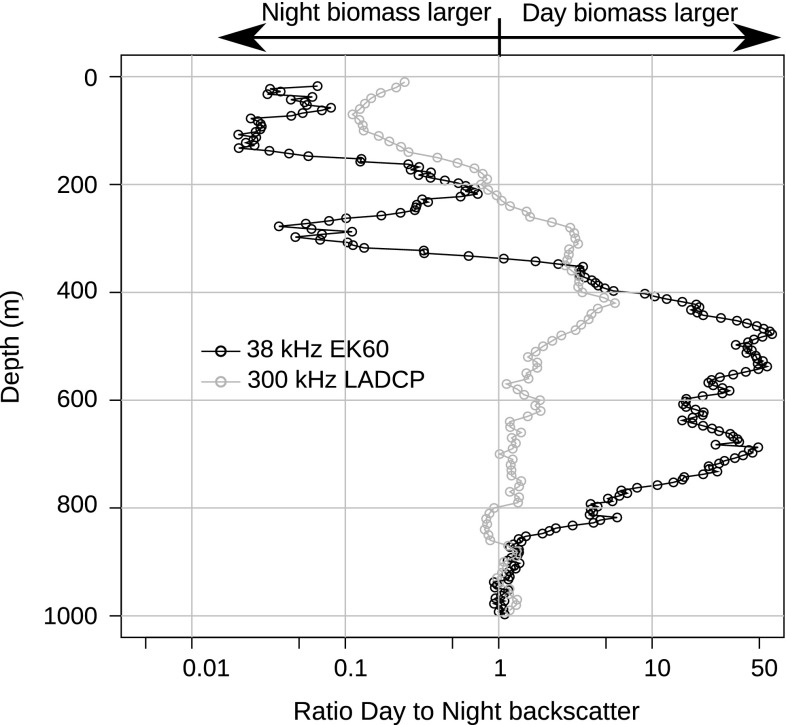
Ratios of day to night acoustic biomass. The *black line* and points show the average ratio between the daytime and night-time 38 kHz EK60 backscatter, integrated in 10 m bins. The *grey line* and points also show the average day-to-night backscatter ratio, but based on the day and night 300 kHz LADCP casts. Note that the plots may be noisy in depth intervals with very low backscattering and that the ratio axis is logarithmic

During the night, the bulk of the 38 kHz acoustic scattering was located in waters shallower than 200 m, with acoustic backscattering often peaking above the fluorescence maximum (Fig. 3). The proportion of migrating animals was very high for the third and fourth layers, and only very weak backscattering was recorded at the depths of these scattering layers during night. To assess vertical shifts of the biomass, we computed the ratio of the daytime acoustic backscatter at a depth to the night-time values at the same depth (Fig. 4). In the interval from 1,000 to ~850 m, the ratio between the day and night in the 38 kHz data is very close to 1, reflecting similar backscattering and, presumably, densities during both day and night. Above this depth, the ratio rapidly increased, and in the interval between ~750 and ~400 m, the average ratio was larger than 10 (i.e. backscattering during the day was more than 10 times higher than during the night). Above 400 m, this ratio declined and, in the upper ~350 m, it was always below 1, reflecting higher nocturnal backscattering, with the ratio ranging from ~1 to 0.02.

The daytime LADCP data were dominated by a single mesopelagic scattering layer, as well as a layer in the epipelagic zone. The vertical distribution of the mesopelagic layer partly overlapped that of the third layer in the EK60 data (Fig. 3). However, the LADCP backscatter peaks consistently occurred ~50 m shallower than the EK60 backscatter peaks and, therefore, in waters with lower oxygen contents. During the night, more of the LADCP backscattering could be found in the upper ~200 m, and a concurrent drop in scattering from the mid-depth layer was evident. Still, a LADCP scattering layer was evident at mesopelagic depths also at night (Fig. 3). The averaged ratio between the day and night backscattering strength was close to 1 at depths below 600 m and showed a peak around 400 m, where the average daytime backscattering strengths were approximately five times higher than the nocturnal values (Fig. 4). In waters shallower than 300 m, the average ratio declined steadily with decreasing depths, passing unity at around 200 m and peaking at a value approaching 0.1, slightly above 100 m.

During dives with underwater vehicles, no larger animals were seen at depths corresponding to layer 1. Around the depths of layer 2, small silver-sided fish, possibly young *Maurolicus sp*. or *Vinciguerria sp*., started appearing in the vehicles’ lights. Deeper down, at depths approximately corresponding to layer 3, the occurrence of what appeared to be myctophid fish was documented, together with larger silver-sided fish. The densities of the fish captured by video strongly suggest that the fish were attracted by the lights on the submersible vehicles. When the vehicles stopped, the numbers of fish rapidly increased. It appeared that the fish were involved in a feeding frenzy as they rapidly darted around. Siphonophores, pelagic shrimps, as well as occasional squid were also observed at these depths. The squid were usually only detectable by their lingering ink-clouds.

## Discussion

We used a hull-mounted 38 kHz EK60 echosounder to map the distribution of scatterers in the Red Sea. Our results suggest that mesopelagic scatterers are abundant down to ~800 m (daytime) throughout the northern Red Sea, bottom depth permitting. Distributions encompassed the OMZ, although the peak backscatter occurred outside the minimum oxygen concentrations. A very high proportion of the mesopelagic organisms detected at 38 kHz carry out DVM. Acoustic targets recorded by a LADCP showed a closer affiliation with the OMZ, they carried out DVM, but a higher proportion remained at mesopelagic depths at night.

### Identification

The animals detectable by the hull-mounted 38 kHz transducer segregated into at least four distinct acoustic layers. A combination of observations from submersible and ROV dives, the acoustic data, as well as previous studies in the Red Sea suggest that mesopelagic fish are an important part of layers 2–4. In a previous study, Dalpadado and Gjøsaeter (1987) found mesopelagic fish in scattering layers at depths of 350 m and deeper during the daytime, but they noted that pearlside (*Maurolicus* *muelleri*) were sometimes found in scattering layers or schools between 50 and 200 m, that is, at the depth range of our layer 2. The other dominant species of mesopelagic fish found by Dalpadado and Gjøsaeter (1987) was *Benthosema pterotum*, which is likely to be found in the deeper layers, where myctophids were observed from the ROV and the submersible.

The incomplete overlap between the scattering layers as seen in the 300 kHz LADCP data and the 38 kHz EK60 data suggests that different organisms were observed with the two instruments. The backscattering strength of any given organism is frequency dependent (Greenlaw 1979), and the higher frequency of the LADCP allows it to detect smaller organisms than the 38 kHz echosounder can detect. The layers picked up by the LADCP therefore likely represent the increased density of meso- and macrozooplankton, which are acoustically invisible to the relatively long wavelength of the hull-mounted 38 kHz transducer. Previous studies have shown that peak daytime mesopelagic mesozooplankton densities coincide with the OMZ (Weikert 1982), which matches well with the vertical distribution of acoustic backscattering identified by the LADCP (Fig. 3). It has been demonstrated that mesopelagic fish may actively avoid lowered systems (Kloser et al. 1997). We interpret the lack of peaks in the LADCP backscatter data at the depths of the EK60 maxima as a result of avoidance from the fairly large instrument package (CTD, rosette with 12, 10-litre Niskin bottles, LADCP), possibly in tandem with the relatively small observation volumes for the LADCP. We consequently hypothesise that the layers observed with the EK60 system consist of animals with active avoidance behaviours and the potential to swim relatively fast. Therefore, based on the acoustic considerations, the visual observations, and previous studies, we propose that layers 3 and 4 consist primarily of mesopelagic fish.

The migration speeds of these layers are similar to previous observations reported for layers of mesopelagic fish in the Arabian Sea (Luo et al. 2000; Ashjian et al. 2002). Both deep layers migrated faster when descending than when ascending, with the descent speeds almost twice as fast. This pattern has previously been described for layers of mesopelagic fish, both in the Arabian Sea and elsewhere (Robinson and Gomez-Gutierrez 1998; Luo et al. 2000; Ashjian et al. 2002). According to Widder and Frank (2001), animals should move faster when they are descending at dawn than when they are ascending during dusk because of their perception of isolumes or preferred light levels. It is also possible that in the morning, when satiated after a night of feeding, these organisms are more risk averse than when they are hungry after daytime digestion and simply opt to swim rapidly down from the rapidly increasing light to the safety of the deep, dark waters.

The highest levels of 38 kHz acoustic backscattering were found in layer 3, where the backscattering strengths peaked in the region 400 to 600 m during daytime. Peak daytime backscattering in the zooplankton layer detected by the LADCP occurred slightly shallower, although there was overlap between these two layers (Fig. 3). This implies that peak daytime acoustic densities were found to be broadly overlapping with the mid-water oxygen minimum zone (Fig. 3). To reach shallow water during nocturnal vertical migrations, the bulk of the biomass in layers 3 and 4 has to migrate through waters which falls under the definition of hypoxic (1.4 ml L^−1^, Ekau et al. 2010), and the vertical extent of this layer may be several hundreds of meters.

Even though the oxygen concentrations of the OMZ are higher in the Red Sea than in, for instance, the Arabian Sea, the high temperatures increase the metabolic rates of the animals (Torres et al. 1979; Donnelly and Torres 1988). In a comprehensive study, Donnelly and Torres (1988) found that for myctophids, the average Q10 was 3.90, meaning that for a 10 °C increase in temperature, the basal metabolism of the myctophids increases by a factor of almost four. The 21.7 °C found in the deep waters of the Red Sea is more than 10 °C higher than in many other mesopelagic environments, suggesting that the oxygen demands of myctophids are more than 4 times higher in the Red Sea than at comparable depths in other oceans. While the oxygen concentrations in the OMZ in the northern Red Sea are not extremely low, the increased oxygen demand caused by the high temperatures effectively increases the oxygen shortage. However, there was no clear evidence that low oxygen waters constrained the distribution of mesopelagic scattering layers. Layer 3, which inhabited the OMZ, stayed below the lowest oxygen concentrations, but we cannot discriminate any effect by hypoxia from, for example, that of light, which is a major factor governing the vertical distribution of mesopelagic scattering layers (Staby and Aksnes 2011).

As the higher temperatures lead to increased metabolic rates for mesopelagic fish, it is possible that these animals may seek to reduce their energy expenditure and oxygen consumption by decreasing their activity levels, as has been documented for other species in other environments (Klevjer and Kaartvedt 2011). Very low activity levels for mesopelagic fish may, however, be more the norm than an exception from a global perspective. Several studies have noted the lethargy of myctophids in particular (Barham 1966; Kinzer et al. 1993, Kaartvedt et al. 2009). Previous studies have shown that in the hypoxic waters of the Arabian Sea, feeding among myctophids is restricted to the night-time (Kinzer et al. 1993). This has been ascribed to the increased abundance of prey in the upper waters, although the low oxygen concentrations experienced by the myctophids at daytime depths may also restrict their metabolic scope for swimming and digestion (Seibel 2011). Even if this small scope for activity during the day has small consequences on the activity levels of myctophids, the constraint is likely to be more important to larger, active piscivores, and the warm, oxygen-deficient waters might therefore create a refuge for the deep-living, smaller planktivorous mesopelagic fish (Prince and Goodyear 2006; Rosa and Seibel 2008), but see (Jorgensen et al. 2009).

### DVM

The most prominent feature in the 38 kHz echograms was the pronounced DVM of the mesopelagic layers. The deepest layer in Fig. 2 migrates about 700 m. The integration results from both acoustic instruments suggest that the biomass and DVM are mainly restricted to the upper 850 m (Figs. 3, 4).

In addition to the high amplitude of the DVM, the DVM in the Red Sea was characterised by the high proportion of biomass taking part in the nocturnal ascent (Figs 2, 3, 4). In both layers 3 and 4, a very high proportion of animals migrate. Our data show that only a marginal proportion of the backscattering remains in the deep during night, average levels were below 3 % in the region 400–600 m, corresponding to layer 3, (Fig. 4) and below 5 % in the region 600–800, corresponding to layer 4. This observation deviates from reports from most oceans, where a considerable proportion of mesopelagic animals usually remain at depth also during the night (Badcock and Merrett 1976; Pearcy et al. 1979; Angel and Pugh 2000; Sutton et al. 2008; Kaartvedt et al. 2009). We tentatively ascribe the high proportion of migrating animals to the warm, oligotrophic waters of the northern Red Sea and the low abundances of prey in the deep waters here when compared to Red Sea surface waters and deep waters in other areas (Wishner 1980; Weikert 1982). The high temperatures would lead to rapid digestion and high metabolic rates, and thus a relatively high demand for food. However, the low prey abundances in the deep waters likely prevent effective daytime feeding, resulting in daily forage migrations to the upper layers. This explanation is not at odds with similar migration patterns in the very productive, low-oxygen areas of the adjacent Arabian Sea, where a very high proportion of the mesopelagic fish layers also appear to migrate. The migration in the Arabian Sea has been ascribed to the low oxygen content forcing a nocturnal ascent to the oxygen-rich surface layers (Kinzer et al. 1993; Morrison et al. 1999).

The LADCP data suggested that some zooplankton remained in the OMZ at night (Figs. 3, 4). Adaptations to low oxygen conditions are common among the mesopelagic biota (Childress and Thuesen 1995; Childress and Seibel 1998; Seibel 2011), and some species are known to inhabit OMZs permanently. Studies have also found that the metabolism of crustaceans is less dependent on the temperature than that of myctophids. Donnelly and Torres (1988) reported a Q10 value of 2.2 for all crustaceans combined, whereas the corresponding value for myctophids was 3.9. In the nearby Arabian Sea, where both oxygen concentrations and temperatures are lower, all larger organisms are reported to leave the OMZ at night (Morrison et al. 1999), while some zooplankton are permanent residents of the low oxygen region just below lower interface of the OMZ, where they possibly may seek refuge from predators by “hiding” in low oxygen waters (Wishner et al. 2000, 2008).

In summary, the acoustic data showed that despite low food concentrations, high temperatures and low oxygen, the mesopelagic zone in the northern Red Sea is abundantly inhabited during daytime. Diel vertical migrations were pronounced, with a very high proportion of acoustically visible mesopelagic animals swimming out of the zone during night. We tentatively ascribe this to low feeding opportunities and fast digestion in the warm waters at mesopelagic depths. Corresponding migration patterns in adjacent, more hypoxic waters have been explained by low oxygen content forcing nocturnal ascents to the oxygen-rich surface layers (Kinzer et al. 1993; Morrison et al. 1999). Predictions suggest that the global oceans will become warmer and less oxygenated (Ramirez-Llodra et al. 2010; Stramma et al. 2010). This underlines the need to better understand how temperature and hypoxia affect the abundance, distribution and behaviour of mesopelagic animals.
